# Rehabilitation needs screening to identify potential beneficiaries: a scoping review

**DOI:** 10.1136/bmjph-2023-000523

**Published:** 2024-04-19

**Authors:** Wouter De Groote, Melissa Corso, Kent Murnaghan, Antony Duttine, Carla Sabariego

**Affiliations:** 1 Department for Noncommunicable Diseases, Rehabilitation and Disability, World Health Organization, Geneva, Switzerland; 2 Ontario Tech University, Oshawa, Ontario, Canada; 3 Canadian Memorial Chiropractic College, Toronto, Ontario, Canada; 4 Faculty of Health Sciences and Medicine, University of Lucerne, Lucerne, Switzerland; 5 Center for Rehabilitation in Global Health Systems, University of Lucerne, Lucerne, Switzerland; 6 Swiss Paraplegic Research, Nottwil, Switzerland

**Keywords:** Public Health, Comorbidity, Demography, Mass Screening

## Abstract

**Objectives:**

The aim is to identify and compare the content of screening tools and needs assessments used to select rehabilitation beneficiaries and to describe the context of their use.

**Design:**

Scoping review.

**Data sources:**

We systematically searched five indexed databases for studies published from 1 January 2010 to 3 February 2023.

**Eligibility criteria:**

We searched for papers published in English only. Papers describe a screening tool or needs assessment aiming to prospectively select potential beneficiaries of rehabilitation services based on a cut-off score or classification system.

**Data extraction and synthesis:**

We charted the evidence according to the characteristics of the paper, rehabilitation needs screening context, screening tool and content of the screening tool. A descriptive synthesis is provided for screening methodology, settings, target populations, rehabilitation need types and phases of care. The WHO International Classification of Functioning, Disability and Health is used to categorise screening items.

**Results:**

We identified 24 tools that use a range of screening methodologies, but mostly questionnaires that are used by health workers. Most tools have been proposed for the identification of a rehabilitation beneficiary among people with selected health conditions assessing the need to access a specific rehabilitation intervention, programme or occupational group. The majority of tools screen for current functioning limitations, and this is often the only screening component. When mapping screening items with the WHO International Classification of Functioning, Disability and Health (ICF), almost all ICF chapters for body functions and activities and participation have been included across screening tools, with the following most frequently included ICF categories: emotional functions (b152), acquiring, keeping and terminating a job (d845), sensation of pain (b280) and carrying out daily routine (d230).

**Conclusions:**

Rehabilitation need screening tools commonly include the screening for current functioning limitations among people with selected health conditions. A screening tool that is applicable across health conditions and settings is not available.

**WHAT IS ALREADY KNOWN ON THIS TOPIC**

- Rehabilitation needs screening is essential to inform the planning and delivery of rehabilitation services at clinical, facility and population levels. To our knowledge, no evidence synthesis on rehabilitation need screening tools has previously been published.

**WHAT THIS STUDY ADDS**

- This study identified 24 rehabilitation need screening tools that have been published from 1 January 2010 to 3 February 2023. Except for the screening for a level of functioning limitations, no other screening component has been commonly used. When mapping with the WHO International Classification of Functioning, Disability and Health, screening items are spread across tools, even across those tools that aim to select a beneficiary among the general population.

**HOW THIS STUDY MIGHT AFFECT RESEARCH, PRACTICE OR POLICY**

- To develop a generic rehabilitation need screening tool that is applicable across health conditions and settings, a valid and evidence-based metric of functioning domains would need to be considered, and additional screening components investigated for optimal sensitivity and specificity.

##  Introduction

Global demographic trends entail a steady and dramatic increase in the number of individuals experiencing limitations in functioning.^1^ Rehabilitation services are essential health services to optimise functioning and reduce disability in individuals with health conditions in interaction with their environment^2^ and hence a critical health strategy to improve individual well-being, population health and societal welfare.^3^ Scientific and clinical research have generated a body of knowledge that strongly supports the use of many interventions for rehabilitation with positive outcomes in various populations and health conditions. Rehabilitation has shown to be effective in improving functioning,^4^,^9^ reducing morbidity, including secondary complications, mortality and healthcare use, including hospital length of stay.^10^,^13^ Rehabilitation likewise increases individuals' participation in education, employment and social life.^14^,^17^ Rehabilitation interventions have also been shown to be cost-effective.^18^,^22^ However, especially in low-income and middle-income countries, rehabilitation is a health strategy that has received little attention so far from potential beneficiaries and among health policymakers, and access to services remains limited.^23 24^

One way to increase political priority, improve service planning and foster an appropriate allocation of human and financial resources to rehabilitation is a sound identification of who would benefit from rehabilitation services. A reliable identification of beneficiaries is key for generating evidence-based and consensus-driven solutions.^25^ It has been suggested to describe a rehabilitation need based on an individual’s self-reported health.^26^ A disagreement in rehabilitation potential, however, can be noted between people’s self-perception, which is influenced by their own awareness about rehabilitation services and their benefits, and health worker or carer assessment.^27 28^ Even among health service sites, significant variations exist when conducting rehabilitation eligibility selection processes.^29^

Although standard for many healthcare needs assessments,^30^ the use of descriptive epidemiological data for healthcare planning is challenging when it comes to rehabilitation. People living with a health condition that is amenable to rehabilitation could potentially benefit from rehabilitation at some point in their lives while rehabilitation services planning at national or regional level requires a precise calculation of the people who would benefit from treatment. In addition, routine collection of information from facilities to estimate local prevalence of disease has shown issues in terms of completeness and quality of data.^31^,^33^ Therefore, to inform policy making and planning for improved access to rehabilitation services as well as to guide clinical decision making, there is a need to clearly describe and identify the potential beneficiaries. This is the first element that drives healthcare planning for rehabilitation. An evidence-based argument that rehabilitation is a good economic and social investment depends on reliably characterising the potential beneficiaries of rehabilitation. This characterisation is key to identify unmet needs, support politically negotiated benchmarks for improving access to rehabilitation services and underpin the added value of rehabilitation in terms of the values of individual well-being and societal welfare.^34^

Using Global Burden of Disease data, a recent work estimated that 2.4 billion people have health conditions that could potentially benefit from rehabilitation.^1^ However, for bridging the gap between services needs and provision, these estimates of rehabilitation needs based on epidemiological data need to be complemented with more accurate measurement. Screening tools with good sensitivity and specificity play an essential role in identifying persons who are in need of specific health services but to date, there is no evidence synthesis about screening tools used in rehabilitation. The objective of this scoping review is therefore to identify and compare the content of rehabilitation need screening tools and assessments used to select rehabilitation beneficiaries and to describe the context of their use. The synthesis can guide stakeholders in selecting screening tools for rehabilitation needs as well as criteria used to select a potential rehabilitation beneficiary. Given the current efforts for strengthening rehabilitation in health systems, this scoping review is also fundamental to evaluate whether new screening tools for rehabilitation are needed and to inform future tool developments. The design of this scoping review is aligned with previously published reviews on screening tools.^35 36^

## Materials and methods

The protocol follows the methodology framework from Arksey and O’Malley^37^ and Levac *et al*.^38^ We report our review according to the Preferred Reporting Items for Systematic Reviews and Meta-Analyses Extension for Scoping Reviews guidelines.^39^

### Research question and concepts

The question that guides this scoping review is ‘what is the content of rehabilitation need screening tools and assessments that are used to select beneficiaries of rehabilitation and what is the context of their use in terms of settings, target populations and phases of care?’

Rehabilitation is defined as a ‘set of interventions designed to optimise functioning and reduce disability in individuals with health conditions in interaction with their environment’.^2^ Interventions for rehabilitation aim to promote or restore an aspect of functioning, compensate for permanent limitations in functioning and to prevent secondary conditions. An individual’s rehabilitation need is defined by having a capacity to benefit from rehabilitation care. This is based on a gap in a person’s health state for which interventions for rehabilitation are available or have been identified; a need is for ‘appropriate’ rehabilitation care and includes cost-effective care.^40^ People may be screened for rehabilitation needs at different settings: at a setting of health services delivery including community-delivered healthcare, at a population or community setting and at a health emergency setting. People may be screened to identify the need for acute, sub-acute or long-term rehabilitation care, which then should be confirmed by a broader rehabilitation assessment.

### Patient and public involvement

Patients and/or the public were not involved in the design, or conduct, or reporting of this research.

### Eligibility criteria

We considered papers that match the following criteria:

#### Scope

Papers describe a screening tool or needs assessment (or its development or testing) aiming to prospectively identify or select potential beneficiaries of rehabilitation services, that is, recommending access to further rehabilitation assessment or referral for a rehabilitation programme, specific intervention or profession type. The tool is used to identify people who are expected to benefit from rehabilitation. Papers that describe tool or assessment components without detailed description of specific screening items or questions have been included, and authors were contacted to share the detailed tool or assessment.

#### Rating system

The screening or needs assessment includes a cut-off score or classification system/rating method that defines the selection of a rehabilitation (intervention) beneficiary, as a way to triage between a need or no need for onward referral or assessment. This enables the exclusion of papers that primarily aim to profile people with rehabilitation needs and allows for the identification of those screening components and items that are used to determine a potential capacity to benefit from rehabilitation.

#### Participants/population

The paper describes a rehabilitation needs screening tool or assessment for people with health conditions (eg, persons with musculoskeletal, neurologic, mental health, cardiovascular and respiratory conditions, cancer) or any type of impairment (eg, persons with visual impairment, hearing impairment) or for a general population (without a description of a health condition or impairment).

The following exclusion criteria for papers have been applied: duplicate, publication type (letter to the editor, position paper, book chapter, conference proceeding or retraction letter), study design (intervention efficacy trial and systematic review) and full text not available.

### Search strategy

We systematically searched the following databases for studies published from 1 January 2010 to 3 February 2023: CINAHL (EBSCO), Cochrane Central Register of Controlled Trials (Ovid), EMBASE (Ovid), MEDLINE (Ovid) and PsycINFO (Ovid). We restricted the search to 13 years because the goal was to get an overview of available (recent) tools and settings of use. An experienced medical information specialist developed and tested the search strategy in collaboration with the other authors. The search strategy was then peer reviewed by a second information specialist (librarian)—which resulted in one suggested revision. In line with scoping review methodology, our search strategy was not restrictive and was constructed using broad and inclusive terminology such as rehabilitation, screening tools and needs assessment. We built the search string using MeSH terms and free-text terms linked with Boolean collectors (AND, OR, NOT). We did not limit to any particular study design. We restricted the search to English and excluded grey literature. The complete search strategy is provided in online supplemental file 1.

### Selection process

The paper selection was completed in two phases:

- Title and abstract screening: search results from all databases have been imported into an online systematic review software called Eppi Reviewer.^41^ After deduplication, two reviewers, a junior physiotherapist doctoral candidate and a senior rehabilitation physician working in public health, used the eligibility criteria to screen titles and abstracts independently. The exclusion criteria have been integrated into the software as a menu of coded criteria that has been developed a priori. To ensure reliability between reviewers, a training exercise was conducted prior to the formal screening. Disagreement was settled using a consensus approach between the two reviewers. If the decision to exclude or include an article was conflictive, a team meeting was held with a third team member to make the final decision.
- Full-text screening: The same two reviewers used the same eligibility criteria to screen the full texts of studies independently for eligibility. Papers were grouped when describing the same screening tool or assessment. Similarly, if the decision to exclude or include an article was conflictive, a team meeting was held with a third team member to make the final decision.

### Data extraction and charting

Data extraction of included papers was conducted individually and in duplicate (20%). Information was extracted using a form with definitions and options for describing the characteristics of the paper, the rehabilitation needs screening context, the screening tool or needs assessment, and the content of screening tool or needs assessment (online supplemental file 2).

### Risk of bias assessment

Risk of bias assessment is not necessary for this scoping review.

### Data synthesis

Descriptive statistics are calculated to characterise the included papers (WHO regions, countries, study design/data source). A descriptive synthesis is provided for screening methodology, settings, target populations, rehabilitation need types and phases of care.

A table is developed to provide an overview of screening components for identified screening tools. For the screening component ‘functioning limitations’, the chapters and categories of the WHO International Classification of Functioning, Disability and Health (ICF)^42^ were used to categorise them. The data are synthesised in a way that we describe commonly used ICF chapters and categories for the identification of rehabilitation beneficiaries.

## Results

### Study selection

**Figure 1:**
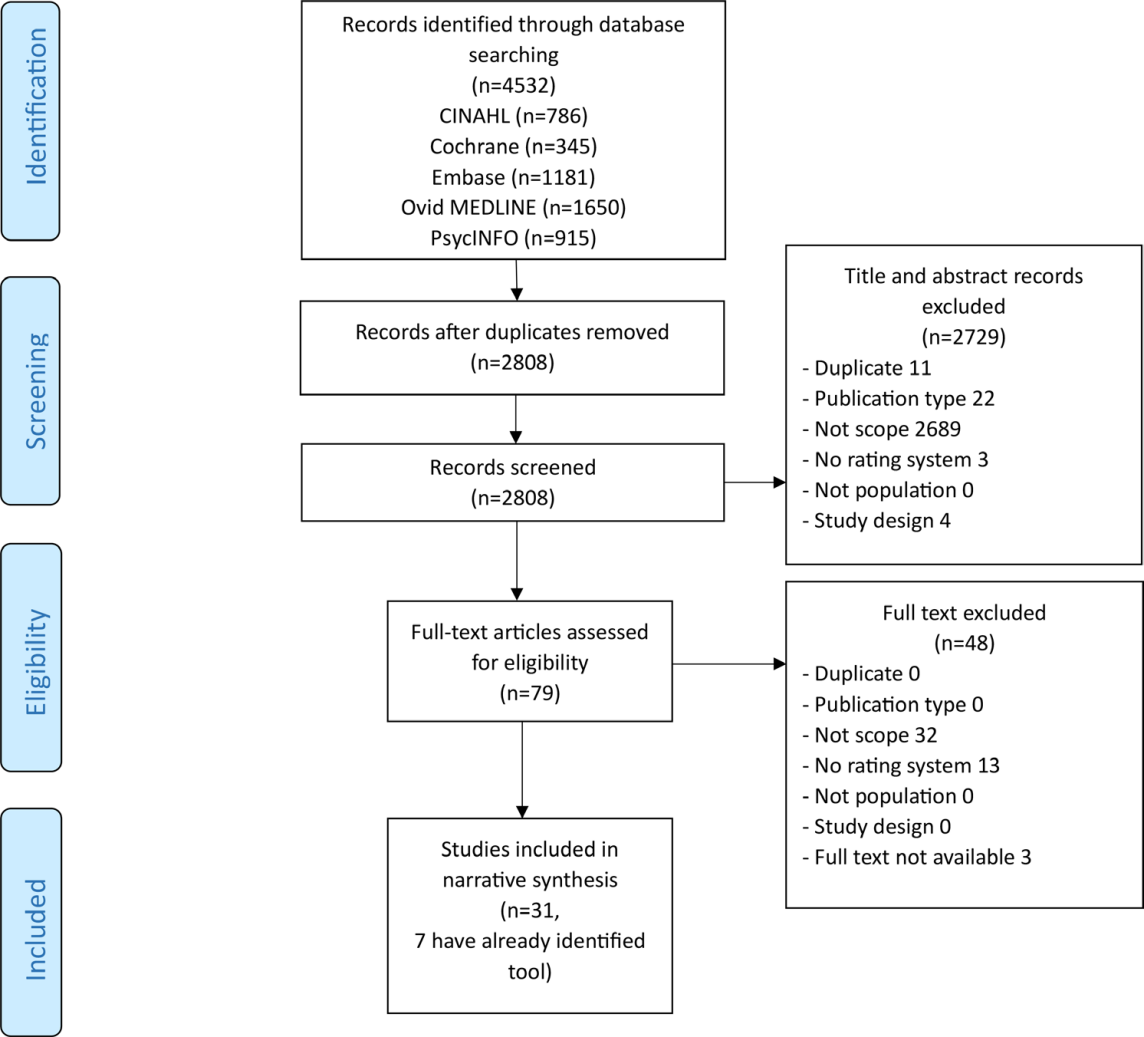
Flowchart.

After duplicate removal, our search strategy identified 2808 papers (figure 1). Out of 79 papers that went through full-text screening, we eventually included 31 articles reporting on a tool or needs assessment.

### Characteristics of included articles

Studies have been identified from across WHO regions, but were predominantly conducted in North America and Europe: African Region (Cameroon, The Gambia, Eritrea), the Eastern Mediterranean Region (Egypt), the South-East Asia Region (India, Thailand), the Region of the Americas (Canada, Chile, USA), the Western Pacific Region (Australia, Singapore) and the European Region (Denmark, Germany, UK, Sweden). In terms of study design, a variety of studies have been included, from observational cross-sectional studies^43^,^51^ and cohort studies,^52^,^58^ over secondary data analysis of population studies,^59^ descriptions of tool development^60 61^ and algorithm development from machine learning,^62^ to prospective observational studies^63^,^66^ and longitudinal studies.^67^,^70^

### Rehabilitation needs screening

In total, 24 rehabilitation need screening tools or assessments have been identified from the 31 included papers. The identified screening tools use a wide range of screening methodologies, mostly original questionnaires,^4748 52^,^54 59 61^ new purpose-made questionnaires,^44 45 50 60 71^ standardised assessment tools,^43 49 62 64^ clinical examinations^46 63^ and scales.^56 65 72^ One tool uses a computer-based algorithm,^57^ while another uses reviews of clinical records.^55^ In most cases a health worker conducts the screening, sometimes requiring direct observation.^43 62 73^ Some scales^72^ and questionnaires^45 52 53 59 71^ are, however, self-administered, or respondents may be care coordinators such as a nurse or a social worker.^44^

The majority of screenings are conducted at a healthcare setting, including at health facilities (level of care not specified),^43 47 71 73^ primary care health centres,^53^ general hospitals,^45 54 60 61 65^ specialised hospitals or clinics,^4649 50 55^,^57 60 63 64 72^ community-delivered healthcare^44 61^ and nursing homes.^47^ Other screenings take place at population or community settings,^48 52 53 59^ including at home.^45 62^ No papers have been identified describing a rehabilitation needs screening at a health emergency setting.

Six tools have been identified that screen for an individual’s rehabilitation need among the general population,^48 49 52 53 59 62^ of which three have been proposed for older people^49 53 62^ and one in a population that is experiencing work ability issues due to a health condition (not specified).^52^ Most tools have been proposed for the identification of a rehabilitation beneficiary among people with specific health conditions or impairments, including children with cerebral palsy,^43 45^ people with a mental health disorder,^44 66^ people with cancer,^45 50 56 60 63 72^ adults with hearing impairment,^71^ athletes,^73^ people with musculoskeletal injuries,^57^ visually impaired children,^46^ stroke survivors,^47^ people with the post COVID-19 condition,^61^ people with central nervous system infections,^64^ critical illness survivors,^65^ people with myotonic dystrophy type 1^49^ and people with lower limb injury^55^ (online supplemental file 3).

The majority of tools are used to screen for a rehabilitation need at the sub-acute to long-term phase of rehabilitation care. Only a few tools have been proposed for the identification of a rehabilitation need at the (sub-)acute phase of rehabilitation care: in critical illness survivors to detect an immediate need for rehabilitation based on a post-intensive care syndrome,^65^ in elderly accessing the hospital to identify a need for comprehensive geriatric assessment including rehabilitation,^54^ and in people with lower limb injury to identify those in need of (sub-)acute inpatient musculoskeletal rehabilitation^55^ (online supplemental file 3).

This review identified a rehabilitation need screening for three specific situations that are integral to rehabilitation practice: for a specific rehabilitation intervention, for a rehabilitation programme or for a rehabilitation occupational group or profession type. When a screening is conducted for the identification of beneficiaries of a specific rehabilitation intervention, all tools are used to identify a need for assistive product provision, such as an adaptive seating system^43^ and other mobility assistive products,^59^ spectacles and low vision devices^46 59^ and hearing aids.^53 59^ A screening for accessing a rehabilitation programme has been used for community delivered rehabilitation,^44 48 60 62^ audiological rehabilitation services,^59 71^ inpatient or outpatient return-to-work rehabilitation,^52 57^ preventative ankle rehabilitation,^73^ post COVID-19 condition personalised rehabilitation,^61^ outpatient or specialised inpatient neurorehabilitation for people with central nervous system infections,^64^ geriatric rehabilitation,^54^ inpatient musculoskeletal rehabilitation^55^ and cancer rehabilitation.^50 56 72^ A need for a referral to a rehabilitation professional involved screening for eligibility of services provided by occupational therapists,^45 49^ physiotherapists,^59 63^ otolaryngologists,^53^ dietitians^48^ and a range of rehabilitation workers for the management of stroke patients and critical illness survivors^47 65^ (online supplemental file 3).

The included papers provide information about sensitivity and specificity of only 10 screening tools and these tools are used across health conditions and rehabilitation need types. Sensitivity ranges from 44% to 93.4% and specificity from 21% to 97%. These accuracy measures have been used to select a cut-off value for their corresponding tools. The other tools did not report their sensitivity and specificity and their thresholds were defined based on clinical judgement, that was sometimes consensus-driven, evidence-based practice, study design or simply following a not further defined choice (online supplemental file 3).

In terms of screening components, 21 (87%) tools screen for current functioning limitations, regardless of whether the screening is carried out in the general population (6/6 tools) or among people with health conditions or impairments (15/18 tools). In these cases, this is often the only screening component (13/21 tools). The remaining tools screen for comorbidity or a decrease in the level of functioning over time. To identify a potential beneficiary of rehabilitation, only a few tools screen for pre-existing functioning limitations (WATT, GSA, ISAR), for basic care support needs (CNS, MST, PICUPS) or for risk factors, such as for functional decline or delayed return to work (WATT, GSA, RALLI). A minority of tools include several screening components simultaneously; CNS, WAI and PICUPS include 3, WATT includes 4, ISAR includes 5 and GSA includes 6 screening components (online supplemental file 4).

Across screening tools for people with specific health conditions, all ICF chapters for body functions and activities and participation have been included for the assessment of limitations in functioning, except for functions of the skin and related structures. ICF chapters of body structures and environmental factors are not usually covered. Among body functions, the ICF chapters on mental functions (8/18 tools), neuromusculoskeletal and movement-related functions (7/18 tools), sensory functions and pain (6/18 tools) and functions of the cardiovascular and respiratory systems (5/18 tools) have been included most frequently. Regarding the ICF activities and participation chapters, tools have mostly covered: mobility (6/18 tools), self-care (6/18 tools), major life areas (5/18 tools), general tasks and demands (4/18 tools) and communication (4/18 tools). When linking screening items to ICF categories of condition-specific screening, these are the categories that are ranked highest, with at least three tools including the screening item: emotional functions (b152), acquiring, keeping and terminating a job (d845), sensation of pain (b280), carrying out daily routine (d230), attention functions (b140), exercise tolerance functions (b455), sensations associated with cardiovascular and respiratory functions (b460), ingestion functions (b510), conversation (d350), transferring oneself (d420) and self-care, unspecified (d599) (online supplemental file 5).

In assessments of functioning limitations in general populations, only a few ICF chapters have been identified for more than one tool: sensory functions and pain (4/6 tools), mental functions (3/6 tools) and mobility (2/6 tools). Interestingly, compared with condition-specific screening, environmental factors such as the availability of assistive products or damaging surrounding sound have been included in one or the other tool.^53 59^ No ICF category could be found that is predominantly used for screening among the general population (online supplemental file 6).

## Discussion

The objective of this scoping review was to identify screening tools and needs assessments for rehabilitation, and to describe their content and context of use. While many more tools and assessments are relevant to rehabilitation practice, such as for diagnosing a condition that is amenable to rehabilitation or assessing the nature of a rehabilitation need to inform and develop an intervention plan, this review focused on tools and assessments that aim to identify an individual that could benefit from rehabilitation services, based on a methodology that selects potential rehabilitation beneficiaries within a given target population. 24 tools have been identified, of which only two were specifically targeting children. The identified tools were mostly questionnaires or standardised assessment tools and less frequently clinical examinations or scales. They were used to identify people in need of either a specific rehabilitation intervention or programme or a rehabilitation professional and, in most cases, were completed by a healthcare worker. Identified tools were commonly implemented at healthcare settings, frequently in specialised hospitals or clinics. Despite the important role of rehabilitation in health emergencies, no tool was used in this context. Most tools were health conditions or impairments specific and used at the sub-acute to long-term phase of rehabilitation care. Although sensitivity and specificity are essential features of any screening tool, only 10 of the included tools provided this information; for the majority of tools thresholds used for deciding on a cut-off indicating a rehabilitation need were defined by clinical judgement or in a further not specified, arbitrary way. Almost 90% of the tools screened for current functioning limitations and in more than half of these, this was the only screening component. Regarding functioning domains, mental functions, neuromusculoskeletal and movement-related functions, sensory functions and pain and functions of the cardiovascular and respiratory systems were the most frequently covered body functions while mobility, self-care and major life areas, which include education and work, were the most frequently screened domains for activities and participation.

Current screening tools for rehabilitation needs do not cover all relevant health condition groups that are amenable to rehabilitation and are restricted to specific settings. This scoping review identified 24 rehabilitation need screening tools from across WHO regions, with a majority of tools aiming to select a potential beneficiary among adults with specific health conditions. Looking at the range of health condition groups in need of rehabilitation, tools have been proposed for musculoskeletal, neurological and mental health conditions, hearing impairment, vision impairment and cancer, but not for respiratory or cardiovascular health conditions, yet these belong to the seven health condition groups with the highest need for rehabilitation.^1^ The appropriateness of the identified tools for health systems steering is also partial. Most of the identified screeners are used in the clinical setting and their focus on specific health conditions is understandable. Nevertheless, for planning and allocating resources to rehabilitation at population level, generic screeners might be more appropriate. From the six identified generic tools, three are targeted at the ageing population, leaving only three with a focus on the general population and suitable to quantify rehabilitation needs at population level. In addition, WHO recently published a policy brief stressing the need for greater preparedness for rehabilitation services in health emergencies, which requires a precise and timely identification of persons in need of rehabilitation.^74^ Nevertheless, our review did not identify any screening tool used in health emergency settings. Finally, available screening tools for rehabilitation are used to select a beneficiary to access an intervention for rehabilitation, a rehabilitation programme or a rehabilitation occupational group, which may be relevant for some health conditions, health systems and funders.^75^ A general screening for rehabilitation needs, however, to be used for instance in primary healthcare triage or to be integrated into routine health surveys is missing.

Health workers are in charge of carrying out screening for rehabilitation needs, mostly using questionnaires. Very often screening is occurring at settings where services have been made available, to raise awareness and increase demand for example, at settings where sub-acute rehabilitation for people with post-intensive care syndrome, community rehabilitation for mental health service users and an occupational therapist for patients with thoracic cancer are available. The screening by health workers using questionnaires is undoubtedly relevant at the clinical level but raises some questions. The value of this relatively easy screening methodology is unknown if information about sensitivity and specificity is lacking, and if cut-off scores are not well justified. Well-established screenings like the ones used in Integrated Care for Older People for identifying declines in functional capabilities in older people at primary care level combine questionnaires with simple clinical tests.^76^ The extent to which questionnaires are sufficient or must be combined with specific tests to precisely identify persons in need of rehabilitation when the broader health system perspective is considered, needs to be investigated. In this review, only a few tools used clinical assessments including direct observations, and it is unclear whether adding such assessments improves the screening outcome for rehabilitation. Given the current very limited funding available for rehabilitation in many countries, a sound screening methodology needs to be used resulting in high levels of both sensitivity and specificity to avoid wasting resources and ensure that rehabilitation planning meets the needs of the population of interest.

Functioning, and its optimisation, is a central goal of rehabilitation and most identified screening tools rely on the overall assessment of functioning or on the assessment of specific functioning domains. The minimal ICF generic set is the universally smallest valid and evidence-based set of functioning domains suitable to track and compare functioning at the clinical and populational levels.^77^ This set encompasses energy and drive functions, emotional functions, sensation of pain, carrying out daily routine, walking, moving around and remunerative employment, and these ICF domains were proved suitable to construct a valid generic functioning metric.^78^ Given that screeners should be as concise as possible and that a valid metric allows for the determination of evidence-informed cut-offs for rehabilitation needs, this set is a good candidate for the development of a screening tool and therefore a good comparator for our results. This scoping review showed that the assessment of functioning domains is spread over the identified tools, including a range of body functions, activity and participation ICF categories. However, many identified condition-specific screening tools encompass only some categories of the minimum generic set while the generic screening tools do not consistently address its domains, pointing out that its evidence was not considered in their development. It is also important to note that despite being central to rehabilitation, information about current functioning limitations may not be sufficient to identify persons who can indeed benefit from rehabilitation. Many tools identified in this review combine the assessment of functioning with further screening components, such as pre-existing functioning limitations, basic care support needs, comorbidities, or the risk of secondary complications, to mention a few. Today, it is impossible to conclude whether having multiple screening components within one tool would increase sensitivity or specificity. Due to the heterogeneity of the identified screeners, and the fact that many studies did not examine sensitivity and specificity, current evidence on which functioning domains are the most suitable for a generic screener as well as about which screening components such a screener should include, besides current functioning limitations, is inconclusive.

In one way or another, a rating method or cut-off score has been defined for all screening tools applying the assessment of functioning limitations to identify a potential beneficiary of rehabilitation. According to Bickenbach *et al*,^34^ limitations in functioning form the foundation of all approaches to characterising a beneficiary of rehabilitation. The authors describe several approaches, from the epidemiologic approach, the human rights characterisation of rehabilitation needs, the reimbursement-based approach to the variety of approaches that use health services as the basis for characterising rehabilitation user groups. In this review, most identified screening tools use an epidemiological approach to identify persons with health conditions for which, in practice, rehabilitation services are effective. Inherent to the definition of a screener, they overcome the main challenge of using the epidemiological approach; defining a threshold of the severity of functioning limitations to select a beneficiary. While screening tools generally include individual items that function as ‘symptoms’ to assess the presence and not the severity of a problem,^68^ screening tools for rehabilitation needs use the level of severity of functioning limitations to determine whether a problem is present or not. The included papers describe several methods for defining thresholds, such as through clinical judgement, trigger questions with yes/no answers, or calculations of cut-offs informed by area under the curve estimations when comparing with scores from clinical assessments to subjectively perceived need. When establishing dichotomous pass–fail scores, screening tool developers make the strong assumption that appropriate interventions for rehabilitation exist to contribute to an improvement of functioning when experiencing a certain level of functioning limitations resulting from a given health condition. Of course, in the attempt of developing a screening tool for the general population, the question remains whether the appropriateness of rehabilitation interventions for a given set of functioning limitations may be extrapolated and therefore applied across health conditions. Also, following the same approach of setting thresholds of the severity of functioning limitations to select a beneficiary, this is accepting that (1) the selection process of a potential rehabilitation beneficiary may change in time as new evidence-based interventions for rehabilitation emerge, (2) an individual’s rehabilitation need may fluctuate over time as a dynamic continuum, depending on the person’s life course and disease progression and (3) the selection process does not take into account the subjectively felt impact on daily life. Regarding the latter, this may result in a demand for rehabilitation that remains low compared with the need, when the lived experience of individuals is not taken into consideration, even in a context of people identifying themselves with rehabilitation needs not necessarily demanding for rehabilitation services.^79^ When bridging the gap between service need and provision for rehabilitation many efforts will be needed that go beyond the identification of an individual’s capacity to benefit from services.

### Limitations

This scoping review has several limitations. We decided not to hand-search reference lists of included papers to only include tools or assessments identified through the systematic search strategy. This may have resulted in not identifying additional papers addressing relevant tools. However, our search strategy detected papers describing the same tool, indicating a good sensitivity of the search strategy. Furthermore, our literature search started with the year 2010 and only included English literature. It is reasonable to assume that older rehabilitation needs screening tools have not been identified. Whether their identification would have impacted our findings is, however, uncertain.

## Conclusions

This overview of identified rehabilitation need screening tools has demonstrated gaps in terms of their potential settings of use and populations in need of rehabilitation. For health system steering, a screening tool that is applicable across health conditions and settings is recommended. This is, however, not yet available, and it is unclear which screening components or functioning domains such a generic screener should have. In the meantime, this synthesis can guide stakeholders developing screening tools for rehabilitation for the general and selected populations in different settings, including for instance primary healthcare or emergencies.

## supplementary material

10.1136/bmjph-2023-000523 online supplemental file 1

10.1136/bmjph-2023-000523 online supplemental file 2

10.1136/bmjph-2023-000523 online supplemental file 3

10.1136/bmjph-2023-000523 online supplemental file 4

10.1136/bmjph-2023-000523 online supplemental file 5

10.1136/bmjph-2023-000523 online supplemental file 6

## Data Availability

Data sharing not applicable as no datasets generated and/or analysed for this study.
