# Supplementary material for: Rehabilitation needs screening to identify potential beneficiaries: a scoping review
Source: BMJ Public Health. 2024 Apr 19;2(1):e000523. doi: 10.1136/bmjph-2023-000523 (PMC11812806; doi:10.1136/bmjph-2023-000523)
Supplement: online supplemental file 1 [file bmjph-2-1-s001.pdf]

Supplementary file 1. Search strategy.

Search Report
Rehabilitation needs screening to identify potential beneficiaries
Time frame: January 1st 2010 to February 3rd 2023

Searches by: Kent Murnaghan
Results: TOTAL: 4532
TOTAL after de-duplication: 2808
Sources: CINAHL (EBSCO), Cochrane Central Register of Controlled Trials (Ovid), EMBASE (Ovid), MEDLINE (Ovid) , and PsycINFO (Ovid).

Search strategies

Source: (OVID) MEDLINE
Hits retrieved (date): 1650 (Feb 3 2023)
Strategy:

1 Rehabilitation/
2 Telerehabilitation/
3 Rehabilitation Centers/
4 Rehabilitation Research/
5 Neurological Rehabilitation/
6 Stroke Rehabilitation/
7 Cardiac Rehabilitation/
8 "Physical and Rehabilitation Medicine"/
9 rehab*.ti,ab,kw.
10 (telerehab* or tele-rehab* or (tele adj2 rehab*)).ti,ab,kw.
11 (rehab* adj2 (need or needs)).ti,ab,kw.
12 (rehab* adj2 (tool* or service*)).ti,ab,kw.
13 rh.fs. [** REHABILITATION]
14 assess*.ti,ab,kw.
15 Needs Assessment/
16 (assess* adj2 (need or needs)).ti,ab,kw.
17 (assess* adj5 (tool* or instrument* or screen* or test* or mechanism* or device* or measur*)).ti,ab,kw.
18 (screen* adj5 (tool* or instrument* or assess* or test* or mechanism* or device* or measur*)).ti,ab,kw.
19 (categor* adj5 (tool* or instrument* or assess* or test* or mechanism* or device* or measur*)).ti,ab,kw.
20 ((systematic* or mass) adj2 screen*).ti,ab,kw.
21 ((evaluat* or determin* or apprais* or discover* or ascertain* or estimat* or calculat* or measur* or detect* or identif*) adj2 (need or needs)).ti,ab,kw.
22 (assess* adj2 (tool* or instrument* or screen* or test* or mechanism* or device*) adj2 rehab*).ti,ab,kw.
23 "Surveys and Questionnaires"/
24 Checklist/
25 Interview/
26 (survey* or questionnair*).ti,ab,kw.
27 (checklist* or interview* or psychometric* or intak* or scale*).ti,ab,kw. [SCREENING TOOLS / NEEDS ASSESSMENT]**

28 1 or 2 or 3 or 4 or 5 or 6 or 7 or 8
29 15 and 28
30 (tool* or instrument* or screen* or test* or mechanism* or device* or measur*).ti,ab,kw.
31 29 and 30
32 22 or 31
33 11 and 14
34 11 and 18
35 9 and 16
36 30 and 35
37 22 or 31 or 33 or 34 or 36
38 13 and 16
39 30 and 38
40 11 and 17
41 11 and 18
42 12 and 16
43 12 and 21
44 23 or 24 or 25 or 26 or 27
45 11 and 44
46 16 or 17 or 18 or 19
47 10 and 16
48 1 and 17
49 2 and 17
50 22 or 31 or 33 or 34 or 36 or 39 or 40 or 41 or 42 or 43 or 45 or 47 or 48 or 49
51 limit 50 to yr="2010 -Current"
52 limit 51 to english language
53 (comment or clinical conference or congress or consensus development conference or editorial or letter or case reports).pt.
54 52 not 53

Source: (Ovid) EMBASE
Hits retrieved (date): 1181 (Feb 3 2023)
Strategy:

1 Rehabilitation/
2 Telerehabilitation/
3 Rehabilitation Center/
4 Rehabilitation Research/
5 Neurorehabilitation/
6 Stroke Rehabilitation/
7 Heart Rehabilitation/
8 Auditory Rehabilitation/ or Cancer Rehabilitation/ or Pulmonary Rehabilitation/ or Speech and Language Rehabilitation/ or Rehabilitation Medicine/ or Psychosocial Rehabilitation/ or Psychiatric Rehabilitation/
9 (rehab* or neurorehab*).ti,ab,kw.
10 (telerehab* or tele-rehab* or (tele adj2 rehab*)).ti,ab,kw.
11 (rehab* adj2 (need or needs)).ti,ab,kw.
12 (rehab* adj2 (tool* or service*)).ti,ab,kw.

13 rh.fs. [**rehabilitation]
14 assess*.ti,ab,kw.
15 Needs Assessment/
16 (assess* adj2 (need or needs)).ti,ab,kw.
17 (assess* adj5 (tool* or instrument* or screen* or test* or mechanism* or device* or measur*)).ti,ab,kw.
18 (screen* adj5 (tool* or instrument* or assess* or test* or mechanism* or device* or measur*)).ti,ab,kw.
19 (categor* adj5 (tool* or instrument* or assess* or test* or mechanism* or device* or measur*)).ti,ab,kw.
20 ((systematic* or mass) adj2 screen*).ti,ab,kw.
21 ((evaluat* or determin* or apprais* or discover* or ascertain* or estimat* or calculat* or measur* or detect* or identif*) adj2 (need or needs)).ti,ab,kw.
22 (assess* adj2 (tool* or instrument* or screen* or test* or mechanism* or device*) adj2 rehab*).ti,ab,kw. [** screening tools / needs assessment]
23 Questionnaire/
24 Checklist/
25 Interview/
26 (survey* or questionnair*).ti,ab,kw.
27 (checklist* or interview* or psychometric* or intak* or scale*).ti,ab,kw.
28 1 or 2 or 3 or 4 or 5 or 6 or 7 or 8
29 15 and 28
30 (tool* or instrument* or screen* or test* or mechanism* or device* or measur* or scale*).ti,ab,kw.
31 29 and 30
32 11 and 14
33 11 and 18
34 9 and 16 and 30
35 13 and 16 and 30
36 11 and 17
37 11 and 18
38 12 and 16
39 12 and 21
40 23 or 24 or 25 or 26 or 27
41 11 and 40
42 10 and 16
43 1 and 17
44 2 and 17
45 22 or 31 or 32 or 33 or 34 or 35 or 36 or 37 or 38 or 39 or 41 or 42 or 43 or 44
46 limit 45 to yr="2010 -Current"
47 limit 46 to english language
48 (books or chapter or conference abstract or conference paper or conference review or editorial or letter).pt.
54 47 not 48

Source: CINAHL (EBSCO)
Hits retrieved (date): 786 (Feb 3 2023)
Strategy:

1. (MH Rehabilitation)
2. (MH "Rehabilitation, Pulmonary") OR (MH "Rehabilitation, Speech and Language") OR (MH "Rehabilitation of Persons with Vision Loss") OR (MH "Rehabilitation of Persons with Hearing Loss") OR (MH "Rehabilitation, Geriatric") OR (MH "Rehabilitation, Cardiac") OR (MH "Rehabilitation, Cancer")
3. (MH "Telerehabilitation")
4. (MH "Rehabilitation Centers")
5. (MH "Research, Rehabilitation")
6. TI rehab* or AB rehab*
7. TI (telerehab* or tele-rehab* or (tele n2 rehab*)) or AB (telerehab* or tele-rehab* or (tele n2 rehab*))
8. TI (rehab* n2 (need or needs)) or AB (rehab* n2 (need or needs))
9. TI (rehab* AND (tool* or service*) or AB (rehab* AND (tool* or service*)
10. MW "rh"
11. TI assess* or AB assess*
12. (MH "Needs Assessment")
13. TI (assess* n2 (need or needs)) or AB (assess* n2 (need or needs))
14. TI assess* n5 (tool* or instrument* or screen* or test* or mechanism* or device* or measur*) or AB assess* n5 (tool* or instrument* or screen* or test* or mechanism* or device* or measur*)
15. TI screen* n5 (tool* or instrument* or assess* or test* or mechanism* or device* or measur*) or AB screen* n5 (tool* or instrument* or assess* or test* or mechanism* or device* or measur*)
16. TI (systematic* or mass) n2 (screen*) or AB (systematic* or mass) n2 (screen*)
17. TI ((evaluat* or determin* or apprais* or discover* or ascertain* or estimat* or calculat* or measur* or detect* or identif*) n2 (need or needs)) or AB ((evaluat* or determin* or apprais* or discover* or ascertain* or estimat* or calculat* or measur* or detect* or identif*) n2 (need or needs))
18. TI (assess* n2 (tool* or instrument* or screen* or test* or mechanism* or device*)) n2 rehab* or AB (assess* n2 (tool* or instrument* or screen* or test* or mechanism* or device*)) n2 rehab*
19. TI (tool* or instrument* or screen* or test* or mechanism* or device* or scale*) or AB (tool* or instrument* or screen* or test* or mechanism* or device* or scale*)
20. (MH "Health Services Needs and Demand")
21. (MH "Surveys")
22. (MH "Questionnaires")
23. (MH "Checklists")
24. (MH "Interviews")
25. TI (survey* or questionnair* or checklist* or interview* or psychometric* or intak* or scale*) or AB (survey* or questionnair* or checklist* or interview* or psychometric* or intak* or scale*)
26. S1 OR S2 OR S3 OR S4 OR S5 OR S6
27. S12 AND S19 AND S26
28. S8 AND S11 AND S19
29. S8 AND S15
30. S6 AND S13 AND S19
31. S10 AND S13 AND S19
32. S8 AND S14
33. S8 AND S15
34. S9 AND S13

35. S9 AND S17
36. S21 OR S22 OR S23 OR S24 OR S25
37. S8 AND S36
38. (MH "Telerehabilitation")
39. S13 AND S38
40. S1 AND S14
41. S14 AND S38
42. S18 OR S27 OR S28 OR S29 OR S30 OR S31 OR S32 OR S33 OR S34 OR S35 OR S37 OR S39 OR S40 OR S41
43. S42 NOT PT (abstract or brief item or book review or case study or commentary or editorial or letter or proceedings)
44. LIMIT S43 English language
45. LIMIT S44 2010-current

Source: (Ovid) Cochrane Central Register of Controlled Trials
Hits retrieved (date): 345 (Feb 3 2023)
Strategy:

1 Rehabilitation/
2 Telerehabilitation/
3 Rehabilitation Centers/
4 Rehabilitation Research/
5 Neurological Rehabilitation/
6 Stroke Rehabilitation/
7 Cardiac Rehabilitation/
8 "Physical and Rehabilitation Medicine"/ or Psychiatric Rehabilitation/
9 (rehab* or neurorehab*).ti,ab,kw.
10 (telerehab* or tele-rehab* or (tele adj2 rehab*)).ti,ab,kw.
11 (rehab* adj2 (need or needs)).ti,ab,kw.
12 (rehab* adj2 (tool* or service*)).ti,ab,kw.
13 rh.fs.
14 assess*.ti,ab,kw.
15 Needs Assessment/
16 (assess* adj2 (need or needs)).ti,ab,kw.
17 (assess* adj5 (tool* or instrument* or screen* or test* or mechanism* or device* or measur*)).ti,ab,kw.
18 (screen* adj5 (tool* or instrument* or assess* or test* or mechanism* or device* or measur*)).ti,ab,kw.
19 (categor* adj5 (tool* or instrument* or assess* or test* or mechanism* or device* or measur*)).ti,ab,kw.
20 ((systematic* or mass) adj2 screen*).ti,ab,kw.
21 ((evaluat* or determin* or apprais* or discover* or ascertain* or estimat* or calculat* or measur* or detect* or identif*) adj2 (need or needs)).ti,ab,kw.
22 (assess* adj2 (tool* or instrument* or screen* or test* or mechanism* or device*) adj2 rehab*).ti,ab,kw.
23 "Surveys and Questionnaires"/
24 Checklist/
25 Interview/
26 (survey* or questionnair*).ti,ab,kw.

27 (checklist* or interview* or psychometric* or intak* or scale*).ti,ab,kw.
28 1 or 2 or 3 or 4 or 5 or 6 or 7 or 8
29 15 and 28
30 (tool* or instrument* or screen* or test* or mechanism* or device* or measur* or scale*).ti,ab,kw.
31 (20 or 29) and 30
32 22 or 31
33 11 and 14
34 11 and (18 or 19)
35 9 and 16
36 30 and 35
37 22 or 31 or 33 or 34 or 36
38 13 and 16
39 30 and 38
40 11 and 17
41 11 and 18
42 12 and 16
43 12 and 21
44 23 or 24 or 25 or 26 or 27
45 11 and 44
46 16 or 17 or 18 or 19
47 10 and 16
48 1 and 17
49 2 and 17
50 22 or 31 or 33 or 34 or 36 or 39 or 40 or 41 or 42 or 43 or 45 or 47 or 48 or 49
51 limit 50 to yr="2010 -Current"
52 limit 51 to english language
53 (comment or clinical conference or congress or consensus development conference or editorial or letter or case reports).pt.
54 52 not 53

Source: (Ovid) PsycINFO
Hits retrieved (date): 915 (Feb 3 2023)
Strategy:

1 rehabilitation/ or neuropsychological rehabilitation/ or neurorehabilitation/ or psychosocial rehabilitation/ or rehabilitation centers/ or telerehabilitation/
2 psychosocial rehabilitation/
3 (rehab* or neurorehab*).ti,ab,tw.
4 (telerehab* or tele-rehab* or (tele adj2 rehab*)).ti,ab,tw.
5 (rehab* adj2 (need or needs)).ti,ab,tw.
6 (rehab* adj2 (tool* or service*)).ti,ab,tw.
7 assess*.ti,ab,tw.
8 needs assessment/
9 (assess* adj2 (need or needs)).ti,ab,tw.
10 (assess* adj5 (tool* or instrument* or screen* or test* or mechanism* or device* or measur*)).ti,ab,tw.
11 (screen* adj5 (tool* or instrument* or assess* or test* or mechanism* or device* or measur*)).ti,ab,tw.

12 (categor* adj5 (tool* or instrument* or assess* or test* or mechanism* or device* or measur*)).ti,ab,tw.

13 ((systematic* or mass) adj2 screen*).ti,ab,tw.

14 ((evaluat* or determin* or apprais* or discover* or ascertain* or estimat* or calculat* or measur* or detect* or identif*) adj2 (need or needs)).ti,ab,tw.

15 (assess* adj2 (tool* or instrument* or screen* or test* or mechanism* or device*) adj2 rehab*).ti,ab,tw.

16 exp surveys/

17 exp "Checklist (Testing)"/

18 interviews/ or focus group interview/ or semi-structured interview/

19 questionnaires/

20 (survey* or questionnair*).ti,ab,tw.

21 (checklist* or interview* or psychometric* or intak* or scale*).ti,ab,tw

22 (tool* or instrument* or screen* or test* or mechanism* or device* or scale*).ti,ab,tw.

23 1 or 2

24 8 and 22 and 23

25 5 and 7 and 22

26 5 and 11

27 6 and 9

28 5 and 10

29 5 and 15

30 6 and 14

31 16 or 17 or 18 or 19 or 20 or 21

32 5 AND 31

33 4 and 9

34 10 and 23

35 3 and 10

36 5 and 12

37 13 and 23

38 3 and 13

39 15 or 24 or 25 or 26 or 27 or 28 or 29 or 30 or 32 or 33 or 34 or 35 or 36 or 37 or 38

40 limit 39 2010-current

41 limit 40 to English language
