# Supplementary material for: Rehabilitation needs screening to identify potential beneficiaries: a scoping review
Source: BMJ Public Health. 2024 Apr 19;2(1):e000523. doi: 10.1136/bmjph-2023-000523 (PMC11812806; doi:10.1136/bmjph-2023-000523)
Supplement: online supplemental file 2 [file bmjph-2-1-s002.pdf]

Supplementary file 2. Variables included in the data extraction form

Variable	Description/instructions
**PMID**	PMID number if available
**Title**	Copy and paste from the paper
**Publication year**	Publication year
**Journal**	Journal name
**Authors**	Enter first three authors' full name
**Country**	Add the country or countries in which the study took place. Use the country's English name, don't use abbreviations
**Study design/data source type**	Copy and paste the study design reported in the paper or type of data source
**Paper's findings**	Copy and paste results or conclusion from the abstract, when most relevant to the screening tool implementation and outcome
**Rehabilitation needs screening context**	
**Target population**	People with health conditions or any type of impairment, or without description of specific health conditions (general population). If described, write the health condition's name or health condition group's name. If patients with several single health conditions are included, add them separated by a comma. For example: diabetes, stroke, hip fracture<br>When screening the general population without description of specific health conditions, write "generic". If the target population is "ageing population" or "fragile people" just add ageing population, fragile people. Specify if age range is available
**Rehabilitation need type**	-Describe the type of rehabilitation need that is targeted by the screening methodology. Match finding with any of the following:<br>. Program: rehabilitation program type (e.g. outpatient, inpatient, community delivered (including home), telerehabilitation, neurorehabilitation, geriatric rehabilitation, musculoskeletal rehabilitation, vocational rehabilitation, …).

	. Profession: rehabilitation occupational group type (e.g. physiotherapist, occupational therapist, physical medicine and rehabilitation doctor, ..)<br>. Intervention: rehabilitation intervention type (e.g. exercise therapy, assistive product provision, …).<br>If there is more than one program, profession or intervention type, separate them by commas.<br>. Not specified
**Phase of rehabilitation care**	Match finding with any of the following: Acute, sub-acute, long-term rehabilitation care, or not specified
**Screening tool or needs assessment characteristics**	
**Tool/assessment name**	Write tool/assessment name and provide short description
**Screening methodology**	Match finding with any of the following: Computer-based algorithm, standardized assessment tool, original questionnaire, new purpose-made questionnaire, semi-structured interview, structured interview, clinical exam, direct observation, scale, review of clinical record, other. If there is more than one screening methodology, separate them by commas
**Setting of screening**	Match finding with any of the following: Healthcare setting (primary care health centre, health facility (level of healthcare not specified), general hospital, specialized clinic or hospital, community delivered health services, other), population setting (community not specified, school, home, ..), health emergency setting (hazard type, including displaced people)
**Screening applicant**	This is the person conducting the screening test. Match finding with any of the following: health worker, caregiver and family (proxy-reported), client (self-administered), data manager/administrative worker, unspecified. If there is more than one screening applicant, separate them by commas

**Rating method/selection criteria**	Describe cut off, rating method or selection criteria that are applied
**Psychometric properties for the screening tool**	Psychometric properties for the screening tool: sensitivity, specificity
**Content of screening tool or needs assessment**	
**Screening components**	Match finding with any of the following: Comorbidity, pre-existing functioning limitations, current functioning limitations (including symptoms and work ability), decrease in level of functioning over time, generic health, health-related QoL, basic care support need, risk factors (e.g. likely decrease of functioning, likely delayed return to work), risk of secondary complications, previous rehabilitation care utilization, other health care use, non-health condition-related factors (e.g. factors delaying discharge), sick leave, rehabilitation need identified by third party, other
**Screening component 'Current functioning limitations'**	Organize and code screening items based on the WHO International Classification of Functioning, Disability and Health (ICF), with ICF linking rules (ICF chapters and categories)
