# Supplementary material for: Rehabilitation needs screening to identify potential beneficiaries: a scoping review
Source: BMJ Public Health. 2024 Apr 19;2(1):e000523. doi: 10.1136/bmjph-2023-000523 (PMC11812806; doi:10.1136/bmjph-2023-000523)
Supplement: online supplemental file 3 [file bmjph-2-1-s003.pdf]

Supplementary file 3. Characteristics of included articles and rehabilitation needs screening context

Author (year), country	Tool/needs assessment	Target population	Rehabilitation need type	Phase of care	Rating system, threshold definition	Paper conclusion
Abdallah (2022), Egypt	Gross Motor Function Classification System Expanded and Revised (GMFCS E&R), and Level of Sitting Scale (LSS)	Cerebral palsy, age 4 - 18	Intervention: adaptive seating system	Long-term care	GMFCS level IV or V and LSS level 1–5 scores concurrently, arbitrary	Approximately 44% of the study participants were in need of an adaptive seating system.
Bekas (2013), United Kingdom	Complex Needs Survey	Users of mainstream secondary mental health services	Program: community recovery and rehabilitation team (CRRT)	Long-term care	Socio-occupational functioning score <5, unmet need score >12, arbitrary	The results were used to invite referrals, create a case-load of 150 and allocate the appropriate resources for the new team.
Bentley (2013), United Kingdom	4 selected items from Sheffield Profile for Assessment and Referral to Care (SPARC) questionnaire	Thoracic cancer patients	Profession; occupational therapist	Sub-acute and long-term care	Score equal or more then 2, arbitrary	Of 540 patients screened, 273 (51 %) reported levels of distress which warranted a full occupational therapy assessment.
Bethge (2012), Bethge (2015), Bethge (2018), Germany	Work Ability Index (WAI)	Generic, white-collar workers with at least half-time employment due to a health condition, women and men aged 45–59 years	Program: a 3-week stay in an inpatient rehabilitation centre and includes the opportunity for outpatient aftercare and graded return-to-work.	Long-term care	WAI score ≤37, based on the highest J value for all indicators of the need for rehabilitation	Predictions of perceived need and an intended request were stronger than those of actual use of rehabilitation services: lower baseline WAI category ratings were associated with higher odds of subjectively perceived need for rehabilitation and intention to request rehabilitation 1 year later.
Boggs (2022), Cameroon, Chile, India, The Gambia, Turkey	Washington Group (WG) question sets	Generic, 2+ years – 50+ years	Program, Profession, and Intervention<br>. Vision: distance glasses, rehabilitation services not specified<br>. Hearing: audiological rehabilitation services and hearing aids<br>. Mobility: physiotherapy and environmental modifications, and Aps including up to 11 mobility APs, such as wheelchairs, prosthetics, sticks/canes and orthotics	Not specified	"Some or worse" difficulty cut-off, moderate and variable sensitivity (44–79) and specificity (73–92) for different domains	At least 60% of people with mild/worse impairments who required referral for rehabilitation/AP services, self-reported "some/worse difficulty", and much fewer reported "a lot/worse difficulty." Using the cut-off "a lot or worse" difficulty would miss the vast majority of people with service needs.
Cheng (2015), Canada	InterRAI Contact Assessment (CA), containing the	Generic, ageing population, frail people, comorbidity	Program, Profession: community delivered rehabilitation (home based), through physical therapy	Sub-acute and long-term	Score from 1 to 5, with higher scores indicating	Inconsistencies between variables considered important for classifying clients who need rehabilitation and

	Rehabilitation Algorithm (RA)		or occupational therapy or a combination of both		higher rehabilitation needs, clinical judgment	those identified in this study based on (rehabilitation) use may indicate a discrepancy in the client characteristics considered relevant in theory versus actual practice.
Demorest (2011), United States of America	Screening Test for Hearing Problems (STHP)	Primarily adults with hearing impairment (whether candidates for a hearing aid or not), but also adults without hearing impairment.	Program: audiologic rehabilitation	Not specified	Failure on either scale: for Communication, a cut-off score of 4 (sensitivity 93.3, specificity 85.9), for Adjustment, a cut-off score of 7 (sensitivity 93.4, specificity 68.7)	The two scales of the Screening Test for Hearing Problems can be used to screen for communication and adjustment problems that warrant a comprehensive rehabilitative assessment.
Dobo (2015), United States of America	Balance Error Scoring System (BESS)	Athletes with ankle injury	Program: preventative ankle rehabilitation program	Sub-acute and long-term	Single leg stance on foam surface cut-off score of 5 (sensitivity 81, specificity 21), and tandem stance on foam surface cut-off score of 3 (sensitivity 81, specificity 47)	Two BESS stances may be used as a quick and inexpensive screening tool for athletes to determine who is most in need of a preventative ankle rehabilitation program to decrease the risk of injury
Gross (2013), Gross (2020), Qin (2016), Canada	Work Assessment Triage Tool (WATT)	People with musculoskeletal injuries	Program: single service provider or specific RTW rehabilitation program	Sub-acute and long-term	Computer based classification algorithm, sensitivity 89, specificity 97	The use of machine learning classification techniques appears to have resulted in classification performance better than clinician decision-making. (cave: follow up publication: ‘Overall accuracy of the WATT declined in a more recent cohort and proved less accurate than human clinical recommendations’)
Gyawali (2018), Eritrea	WHO Prevention of Blindness (WHO PB) program form for the recording of children with blindness and vision impairment.	Visually impaired children	Intervention: spectacles and low vision devices (LVDs)	Sub-acute and long-term care	At least one line improvement in distance or near Visual Acuity with refractive correction and/or LVDs, evidence based practice	A significant number of children at the school for the blind benefited from refractive correction and LVDs. With such optical intervention, many of these children could study at mainstream schools with print media.
Kasemsiri (2015), Thailand	Five-Minute Hearing Test (FMHT)	Generic, age 60 and above	Profession, Intervention: audiometry (otolaryngologist) and hearing technology provision	Sub-acute and long-term	Cut-off score of equal to or greater than 12, sensitivity 79.3, specificity 76.0	The public health service should be encouraged to conduct the Thai-FMHT to improve the accessibility of hearing screening
Kjork (2022), Turner (2019), Sweden, United Kingdom	Post-Stroke Checklist (PSC)	Stroke survivors, nursing home residents	Profession: health worker with knowledge about post stroke rehabilitation and rehabilitation workers (PRM, OT, PT, SLT, psychologist)	Sub-acute and long-term	Yes/no trigger questions, clinical judgement	Among the health problems identified with the checklist, activities of daily living (82%) were most common, and spasticity (41%) and pain (29%) were least common.

Lai (2016), United States of America	Prospective Surveillance Model (PSM)	Postoperative breast cancer patients	Profession, Intervention: Physical therapy including manual therapy and soft tissue massage, treatment of cording, targeted home exercise, and/or lymphedema treatment.	Sub-acute and long-term	Lymphedema >3 %, increase in arm circumference >1 cm from baseline, shoulder range of motion decreased by 20, clinical judgement	Patients with more activity restriction and lower levels of function in the early postoperative period may benefit from rehabilitation interventions.
Leipold (2018), Australia	Malnutrition Screening Tool (MST)	Generic, 18 years and above	Program, Profession: Community Rehabilitation Program, dietitian	Sub-acute and long-term	Two categories; 'at risk of malnutrition' or 'not at risk of malnutrition', sensitivity 72.2, specificity 83.8	The main finding is that the MST is a valid tool to screen for malnutrition in a population.
Lethborg (2014), Australia	Geriatric Screening Assessment (GSA)	Older people with cancer	Program, Profession; community-based rehabilitation, with social worker, occupational therapist, physiotherapist, dietitian, speech therapist	Sub-acute	Yes/no trigger questions, clinical judgement	Although this project did not result in referrals of older people with cancer to subacute ambulatory care services (SACS) the training program for staff was a success and allied health assessments were improved to include geriatric screening assessment factors.
Manhas (2022), Canada	Post COVID-19 rehabilitation screening tool (based on the Post COVID-19 Functional Status scale)	Post COVID-19 population	Program: targeted or personalized rehabilitation	Sub-acute and long-term	No services/ self-management (score 0-1), rehabilitation (score 2-4), clinical judgement	The Provincial Post COVID-19 Rehabilitation Response Framework (PCRF) is a framework for health systems to ensure consistent identification, assessment, and management of the rehabilitation needs of post-acute and chronic PCC.
Olsson (2020), Olsson (2022), Sweden	Distress Thermometer	Women following primary breast cancer treatment	Program: individualized cancer rehabilitation plan	Sub-acute and long-term	Score of ≥ 5, based on preliminary analyses of the randomization procedure	This study will provide important knowledge related to effectiveness of screening-based identification of rehabilitation needs and standardized evidence-based, individualized rehabilitation after primary breast cancer treatment.
Orum (2021), Denmark	Montreal Cognitive Assessment (MoCA)	People with central nervous system (CNS) infections	Program: outpatient or specialized inpatient neurorehabilitation	Sub-acute and long-term	Cut-off value of < 26, higher odds of needing rehabilitation based on clinical judgement	As a screening tool, MoCA was able to identify patients in need of rehabilitation. In young adults, MoCA scores were often normal despite a need for rehabilitation.
Puthucheary (2022), Turner-Stokes (2022), United Kingdom	Post-ICU Presentation Screen (PICUPS) (plus) tool	Critical illness survivors at step down from ICU (PICUPS) and at acute care phase (PICUPS plus)	Profession: specific rehabilitation occupational groups (PRM, OT, PT, SLT, psychologist, dietitian)	Acute and sub-acute	Each item (0-5 score) has a cut off score that triggers referral to specific rehabilitation	The PICUPS tool is feasible to implement as a screening mechanism for post-intensive care syndrome and need for rehabilitation.

					occupational groups, clinical judgement	
Raymond (2015), Canada	Pinch gauge and Jamar dynamometer	People with myotonic dystrophy type 1 (DM1), adult and late-onset phenotypes	Program: Rehabilitation services for household activities (occupational therapy)	Sub-acute and long-term	Lateral pinch strength 4.8 kg (sensitivity 75.6, specificity 79.2), grip strength women 6.6 kg (sensitivity 77.5, specificity 71.4), men 3.8 kg (sensitivity 82.1, specificity 82.6)	Potential indicator of needs related to household activities for rehabilitation services with valid assessment tools were developed for people with DM1 who experience difficulties in housing-related activities.
Rosted (2014), Denmark	Identification of Seniors at Risk (ISAR)	Generic, older people	Program: Geriatric interventions and rehabilitation	Acute and sub-acute	Score of ≥ 2, sensitivity 73, specificity 51 (McClusker J, et al. Academic Emerg Med, 2000)	To identify elderly patients with a need for comprehensive geriatric assessment, we recommend that triage be supplemented with the ISAR screening
Rouleau (2015), Canada	Rehabilitation After Lower Limb Injury (RALLI)	People with lower limb injury (fracture or clinically important soft tissue injury affecting structures from the pelvis to the toes)	Program: Inpatient musculoskeletal rehabilitation	Acute and sub-acute	Score of 2, based on risk of rehabilitation uptake (79% to 86%)	We created a predictive score using the top 3 risk factors. Rehabilitation planning should begin for patients exhibiting at least 2 of 3 risk factors at the time of admission to acute care.
Yadav (2016), India	Performance Status Scale for Head and Neck scale (PSS HN)	Oral cancer survivors	Program: Postoperative rehabilitation	Sub-acute and long-term	Score < or equal to 50, based on comparative study (List MA, et al. Cancer, 1996)	Prevalence of functional deficit of eating in public, deficit of understandability of speech and deficit of normalcy of diet were 28, 13 and 38 % respectively
Yen (2022), Singapore	Cancer Rehabilitation Questionnaire (CRQ)	Cancer survivors	Program: Cancer rehabilitation, ranging from exercise-based interventions to cognitive behavioral therapy and cognitive therapy	Sub-acute and long-term	Cut-off score of ≥ 4, sensitivity 61.8, specificity 75.5	The CRQ can help to stratify cancer survivors requiring further rehabilitation interventions.
