# Supplementary material for: Rehabilitation needs screening to identify potential beneficiaries: a scoping review
Source: BMJ Public Health. 2024 Apr 19;2(1):e000523. doi: 10.1136/bmjph-2023-000523 (PMC11812806; doi:10.1136/bmjph-2023-000523)
Supplement: online supplemental file 4 [file bmjph-2-1-s004.pdf]

Supplementary file 4. Rehabilitation need screening tools/assessments and corresponding screening components

Tool/needs assessment	Comorbidity	Pre-existing functioning limitations	Current functioning limitations	Decrease in level of functioning over time	Generic health	Basic care support need	Risk factors	Sick leave	Risk of secondary complications	Other health care use	Need identified by third party
Gross Motor Function Classification System Expanded and Revised (GMFCS E&R), and Level of Sitting Scale (LSS)			✓								
Complex Needs Survey (CNS)			✓			✓					✓
4 selected items from Sheffield Profile for Assessment and Referral to Care (SPARC) questionnaire			✓	✓							
Work Ability Index (WAI)	✓		✓					✓			
Washington Group (WG) question sets			✓								
InterRAI Contact Assessment (CA)			✓								
Screening Test for Hearing			✓								

Problems (STHP)								
Balance Error Scoring System (BESS)		✓						
Work Assessment Triage Tool (WATT)	✓	✓		✓		✓		
WHO Prevention of Blindness program form for the recording of children with blindness and vision impairment.		✓						
Five-Minute Hearing Test (FMHT)		✓						
Post-Stroke Checklist (PSC)			✓					
Prospective Surveillance Model (PSM)			✓					
Malnutrition Screening Tool (MST)		✓			✓			
Geriatric Screening Assessment (GSA)	✓	✓	✓			✓	✓	✓
Post COVID-19 rehabilitation screening tool (based on the		✓						

Post COVID-19 Functional Status scale)								
Distress Thermometer			✓					
Montreal Cognitive Assessment (MoCA)			✓					
Post-ICU Presentation Screen (PICUPS) (plus) tool			✓		✓		✓	
Pinch gauge and Jamar dynamometer			✓					
Identification of Seniors at Risk (ISAR)	✓	✓	✓	✓				✓
Rehabilitation After Lower Limb Injury (RALLI)	✓					✓		
Performance Status Scale for Head and Neck scale (PSS HN)			✓					
Cancer Rehabilitation Questionnaire (CRQ)			✓					
