# Supplementary material for: Rehabilitation needs screening to identify potential beneficiaries: a scoping review
Source: BMJ Public Health. 2024 Apr 19;2(1):e000523. doi: 10.1136/bmjph-2023-000523 (PMC11812806; doi:10.1136/bmjph-2023-000523)
Supplement: online supplemental file 5 [file bmjph-2-1-s005.pdf]

Supplementary file 5. ICF chapters and categories included in health condition-specific rehabilitation need screening tools

ICF chapters	GMFCS E&R and LSS	CNS	SPARC	STHP	BESS	WATT	WHOPB	PSC	PSM	GSA	PCFS	Distr. Therm.	MoCA	PICUPS	Pinch gauge, Jamar dyn.	RALLI	PSSHN	CRQ
**Body Functions**																		
Mental Functions			b130	b152		b152		b152, b140, b144, b160		b130, b152, b140, b144, b164, b167		b152	b114, b140, b144, b164,	b114, b147, b152				
Sensory Functions and Pain						b280	b210	b280			b250, b255, b280			b280				b280
Voice and Speech Functions														b310			b310	
Functions of the Cardiovascular, Haematological, Immunological and Respiratory Systems									b435	b460	b450, b455			b440, b450, b455, b460				b435, b455, b460
Functions of the Digestive, Metabolic, Endocrine Systems										b510, b525, b530				b510			b510	b510
Genitourinary and Reproductive Functions								b620, b525										
Neuromusculoskeletal and Movement-Related					b715			b735	b710	b755				b760	b730			b710, b730,

Functions											b755, b770
Functions of the Skin and Related Structures											
**Body Structures**											
Structure of the Nervous System											
The Eye, Ear and Related Structures											
Structures Involved in Voice and Speech											
Structure of the Cardiovascular, Immunological and Respiratory Systems									s430		
Structures Related to the Digestive, Metabolic and Endocrine Systems						s520, s530,					
Structure Related to Genitourinary and Reproductive Systems											
Structure Related to Movement						s710				s730, s740, s750	
Skin and Related Structures											
**Activities and Participation**											
Learning and Applying Knowledge						d140					
General Tasks and Demands		d230		d230			d230				d230
Communication			d350			d350		b330	d350		
Mobility	d415, d465			d451	d420, d450	d420, d450,			d410, d415,		d410

						d451, d470		d420, d460	
Self Care		d599		d510, d540	d599	d510, d530, d540, d550	d599	d520	
Domestic Life		d640		d620		d620, d630, d640			
Interpersonal Interactions and Relationships			d720		d798				
Major Life Areas	d845, d910			d845	d845	d845	d845		
Community, Social and Civic Life					d920				d910
**Environmental Factors**									
Products and Technology									
Natural Environment and Human-Made Changes to Environment									
Support and Relationships						e310, e340		e310	
Attitudes									
Services, Systems and Policies									
