# Supplementary material for: Rehabilitation needs screening to identify potential beneficiaries: a scoping review
Source: BMJ Public Health. 2024 Apr 19;2(1):e000523. doi: 10.1136/bmjph-2023-000523 (PMC11812806; doi:10.1136/bmjph-2023-000523)
Supplement: online supplemental file 6 [file bmjph-2-1-s006.pdf]

Supplementary file 6. ICF chapters and categories included in generic rehabilitation need screening tools

ICF chapters	Work Ability Index (WAI)	Washington Group (WG) question sets	InterRAI Contact Assessment (CA)	Five-Minute Hearing Test (FMHT)	Malnutrition Screening Tool (MST)	Identification of Seniors at Risk (ISAR)
**Body Functions**						
Mental Functions	b110, b152		b164			b144
Sensory Functions and Pain		b140, b144, b210, b230		b230	b130	b210
Voice and Speech Functions						
Functions of the Cardiovascular, Haematological, Immunological and Respiratory Systems						
Functions of the Digestive, Metabolic, Endocrine Systems					b530	
Genitourinary and Reproductive Functions						
Neuromusculoskeletal and Movement-Related Functions						
Functions of the Skin and Related Structures						
**Body Structures**						
Structure of the Nervous System						

The Eye, Ear and Related Structures			
Structures Involved in Voice and Speech			
Structure of the Cardiovascular, Immunological and Respiratory Systems			
Structures Related to the Digestive, Metabolic and Endocrine Systems			
Structure Related to Genitourinary and Reproductive Systems			
Structure Related to Movement			
Skin and Related Structures			
**Activities and Participation**			
Learning and Applying Knowledge			
General Tasks and Demands		d230	
Communication			d310
Mobility	d450, d451, d465	d450, d451	
Self Care		d510, d520, d540	
Domestic Life		d640	
Interpersonal Interactions and Relationships			

Major Life Areas	d845, d850			
Community, Social and Civic Life				d910
**Environmental Factors**				
Products and Technology		e115, e120, e125		
Natural Environment and Human-Made Changes to Environment				e250
Support and Relationships				
Attitudes				
Services, Systems and Policies				
